# Oligodendrocyte Progenitors in Glial Scar: A Bet on Remyelination

**DOI:** 10.3390/cells13121024

**Published:** 2024-06-12

**Authors:** Davide Marangon, Juliana Helena Castro e Silva, Valentina Cerrato, Enrica Boda, Davide Lecca

**Affiliations:** 1 Laboratory of Molecular and Cellular Pharmacology of Purinergic Transmission, Department of Pharmaceutical Sciences, Università degli Studi di Milano, 20133 Milan, Italy; davide.marangon@unimi.it (D.M.); juliana.castro@unimi.it (J.H.C.e.S.); 2 Department of Neuroscience Rita Levi-Montalcini, University of Turin, 10126 Turin, Italy; valentina.cerrato@unito.it (V.C.); enrica.boda@unito.it (E.B.); 3 Neuroscience Institute Cavalieri Ottolenghi, Regione Gonzole 10, 10043 Orbassano, Turin, Italy

**Keywords:** remyelination, oligodendrocytes, glia, astrocytes, proteoglycans, spinal cord injury, multiple sclerosis, demyelination, CNS lesion

## Abstract

Oligodendrocyte progenitor cells (OPCs) represent a subtype of glia, giving rise to oligodendrocytes, the myelin-forming cells in the central nervous system (CNS). While OPCs are highly proliferative during development, they become relatively quiescent during adulthood, when their fate is strictly influenced by the extracellular context. In traumatic injuries and chronic neurodegenerative conditions, including those of autoimmune origin, oligodendrocytes undergo apoptosis, and demyelination starts. Adult OPCs become immediately activated; they migrate at the lesion site and proliferate to replenish the damaged area, but their efficiency is hampered by the presence of a glial scar—a barrier mainly formed by reactive astrocytes, microglia and the deposition of inhibitory extracellular matrix components. If, on the one hand, a glial scar limits the lesion spreading, it also blocks tissue regeneration. Therapeutic strategies aimed at reducing astrocyte or microglia activation and shifting them toward a neuroprotective phenotype have been proposed, whereas the role of OPCs has been largely overlooked. In this review, we have considered the glial scar from the perspective of OPCs, analysing their behaviour when lesions originate and exploring the potential therapies aimed at sustaining OPCs to efficiently differentiate and promote remyelination.

## 1. Introduction

Responses to injury include a wound-healing structural remodelling of the lesion site and nearby regions, culminating in the formation of a glial scar. This process has been observed in a wide range of CNS pathological conditions, including spinal cord injury (SCI), traumatic brain injury (TBI), ischemic stroke, and neurodegenerative diseases, such as multiple sclerosis (MS). The size and features of the glial scar vary in distinct CNS diseases and injuries, depending on the different pathological dynamics, location and severity of the insult. Yet, a fundamental arrangement of the glial scar can be recognised in all cases, regardless of the primary trigger, and includes three main compartments: a fibrotic lesion core encircled by a compact border of a heterogeneous population of glial cells and, more externally, by an adjacent reactive neural parenchyma [1,2].

The lesion core or fibrotic scar is an area of severe tissue damage—often containing a central cystic cavity—populated by blood-borne cells (i.e., macrophages and lymphocytes), perivascular-derived macrophages and stromal cells. Early after acute injuries, blood-derived cells are recruited to the lesion site by resident astrocytes and microglia—which are the first responders to insults—secreting pro-inflammatory cytokines and chemokines. Then, over time, the stromal component (i.e., fibroblasts and pericytes) progressively prevails over the blood-derived inflammatory cells and becomes the dominant cell population of the fibrotic scar, being embedded in a rich deposit of extracellular matrix (ECM) [3,4]. Newly generated astrocytes, oligodendrocyte progenitor cells (OPCs, or NG2 glia) and microglia are, instead, the main components of the surrounding ring, the proper glial scar [2,5], whereas the adjacent neural parenchyma comprises neurons actively engaged in neurite and synaptic remodelling [6].

Formation of the glial scar has been traditionally interpreted as a protective process, instrumental in confining the inflammatory response and blood-derived cells to the lesion core; however, in the long term, it also contributes to inflammation, chronic damage and impaired repair. The protective role has been mainly attributed to astrocytes and microglia, whose ablation/manipulation leads to an increased influx of blood-derived macrophages and fibrotic cells and exacerbated neuronal cell death, axon loss and demyelination after injury [2,7,8]. The role of the other glial cell types, and particularly of oligodendroglia, participating in the formation of the glial scar has been less investigated so far. Yet, an emerging corpus of data has started to become available. In this review, we will specifically focus on OPCs and oligodendrocytes, their behaviour and their molecular alterations in a non-permissive inflammatory microenvironment, and the potential therapeutic strategies to restore their functions and promote lesion resolution in both acute and chronic conditions.

## 2. Myelin as a Structural and Trophic “Organ” for Axons

In the CNS, oligodendrocytes are highly specialised cells responsible for the synthesis of myelin, a multilamellar structure characterised by up to 70% in lipid and myelin proteins that enable membrane compaction in a sheath that enwraps single axons, thus allowing fast saltatory conduction [9].

The composition of myelin is unique compared to other membranes, with an approximate 2:2:1 ratio for cholesterol/phospholipids/glycolipids [10]. The extremely high cholesterol content in myelin, which represents 80% of the cholesterol present in the brain, is ensured by both the de novo biosynthesis and the uptake of cholesterol precursors from other glial cells [11]. Other lipids are synthesised using fatty acids as building blocks. Myelination is a dynamic process that requires continuous maintenance of the membranes, thus making the oligodendrocytes highly active in both protein and lipid metabolism.

Moreover, in the last decades, it has been clearly demonstrated that oligodendrocytes also provide axons with fuel to enable proper neuronal functions, thus further increasing their energy demand (for review, see Saab and Nave 2017 [12]). Oligodendrocytes can directly transfer glucose through the GLUT1 transporter; in addition, oligodendrocytes metabolise glucose to pyruvate through glycolysis, convert pyruvate to lactate, and then export lactate via monocarboxylate transporter 1 (MCT1) to the axons. Oligodendrocytes could also play a more indirect trophic role, fuelling axons with lactate produced by astrocytes and imported through gap junctions rather than synthesising lactate by extensive glycolysis [13]. Via the gap junctions, oligodendrocytes also regulate the concentrations of ions, such as Na+, potassium K+ and chloride Cl−, in both intracellular and extracellular spaces in the neuron-oligodendrocyte synapse, thus contributing to the maintenance of the resting membrane potential [14,15,16]. More recently, extracellular vesicles have been described as important components that are able to shuttle proteins, lipids, nutrients and non-coding RNAs to axons and also maintain their homeostasis in nutrient-deprived conditions [17]. Axonal trophic support includes typical fatty acids, such as docosahexaenoic acid, which constitutes a major component of the excitable membranes produced through beta-oxidation by oligodendrocyte peroxisomes in the myelin sheath [18].

## 3. More Than Oligodendrocyte Progenitor Cells

Oligodendrocytes originate from OPCs, a population of highly proliferative glia that represents approximately 5–8% of the total cells in the CNS. OPCs derive from multiple niches in the developing brain, and they migrate in both the brain and spinal cord, where they differentiate and contribute to myelination [9] (for extensive review, see [19,20]). OPCs can be identified based on their expression of the platelet-derived growth factor receptor α (PDGFR-α), the proteoglycan NG2. In this early stage, OPCs show small capacitance, currents mediated by K_V_ channels, Na_V_ channels and glutamate receptors [21,22,23]. As OPCs differentiate, they firstly form pre-myelinating oligodendrocytes, downregulating PDGFR-α, NG2, and voltage-gated Na^+^ and Ca^2+^ channels, while they progressively express the sulfatide O4, the galactocerebroside GalC, and GPR17, a G protein-coupled receptor typical of this intermediate stage acting as a transient inhibitor of myelination [24]. During their maturation, pre-myelinating oligodendrocytes progressively downregulate GPR17 and modify their glucose and lipid metabolism to produce myelin proteins and lipids that allow the organisation of the myelin sheath around axons (for review, see [25]). This three-stage classification is an oversimplification since OPCs and oligodendrocytes represent a continuum [26]. Recent transcriptomic analysis in oligodendroglial cells isolated from different brain areas in young and adult mice has identified 13 distinct populations that represent different oligodendroglial functional states [27]. This study highlighted that differentiation-committed oligodendrocyte precursors are distinct from OPCs and show lower levels of cell cycle markers while expressing genes involved in migration. One population of newly formed oligodendrocytes was shown to respond to motor learning, while another of vascular and leptomeningeal cells could migrate by following vessels. Similar results were found in human white matter brain samples [28]. In this study, oligodendroglia were divided into eight subclusters, including OPCs, committed oligodendrocyte precursors, and six types of oligodendrocytes, all expressing myelin protein transcripts, such as MBP and MOG, but with different transcriptional states. This heterogeneity could be both intrinsic to their developmental origin and acquired by extrinsic cues at their final location. It was demonstrated that OPCs change their differentiation ability and show different responsiveness to growth factors when transplanted into other CNS areas [29]. OPCs are the only glial cell type that receives neuronal synapses, whose activity was shown to modulate their proliferation, differentiation and migration and is important for myelination and remyelination (for review, see [30,31]). Recently, OPCs have been shown to phagocytose synapses in the developing and mature mouse visual cortex, thus contributing to the remodelling of neural circuitries [32]. These data highlight that OPCs are extremely plastic and adapt their program in response to a multitude of physiological signals [33].

## 4. Oligodendroglial Cell Responses to Injury

**Figure 1:**
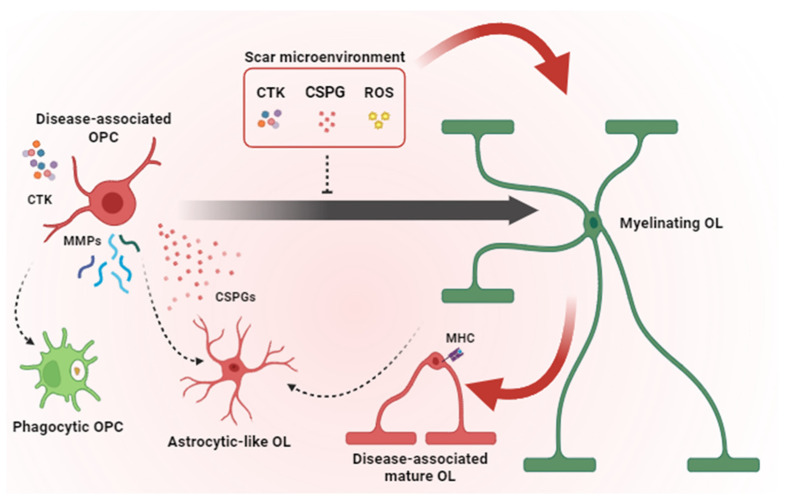
Pathological mechanisms contributing to remyelination failure in glial scar. Microglia and astrocytes contribute to the scar microenvironment by secreting pro-inflammatory CTKs, CSPGs and ROS that inhibit OPC differentiation and induce a disease-associated phenotype in OPCs and mature OLs. Disease-associated OPCs overexpress MMPs, release CSPGs and acquire phagocytic functions and astrocyte-like phenotype instead of generating new myelinating OLs. Together, all these pathological mechanisms lead to remyelination failure. OPC = oligodendrocyte precursor cells; OL = oligodendrocyte; CTK = cytokines; CSPG = chondroitin sulphate proteoglycans; ROS = radical oxygen species; MMP = matrix metalloproteinases; MHC = major histocompatibility complex. Dashed arrows indicate cell conversions. Red arrows point to events which are detrimental to remyelination. The black arrow indicates OPC maturation (i.e., transition toward a myelinating OL state).

Myelin and oligodendrocyte loss start immediately after acute injuries, such as SCI or TBI (i.e., within 15 min and for 24–48 h at the core of the injury), likely due to the combinatorial effects of bleeding, hypoxia, oxidative damage, ATP- and glutamate-mediated excitotoxicity and the release of pro-inflammatory cytokines such as interleukin-1α (IL-1α), and tumour necrosis factor-α (TNF-α) [6,34] (Figure 1). While neurons and astrocytes do not typically die beyond 24 h post-injury, oligodendrocyte apoptosis is instead protracted up to the subacute (i.e., 3–14 days after injury) and chronic phases, especially in distal degenerating axon tracts after SCI [35]. By impairing axonal conduction and reducing the metabolic supply and ion buffering, such a protracted loss of oligodendrocytes and demyelination contribute to neuronal and circuit dysfunction, leading to post-injury functional impairment. Accordingly, the number of spared axons *per se* does not always predict functional recovery, and in the case of SCI, a complete loss of motor and sensory functions frequently also occurs in the presence of anatomically incomplete spinal lesions [34].

While demyelination is still ongoing, OPCs are rapidly recruited at the lesion site and set up a robust proliferative response, peaking at around 5–7 days post-injury in the tissue directly bordering the lesion upon SCI or stroke [34,36,37]. Such OPC expansion can be sustained by both parenchymal OPCs and periventricular neural stem cells [38,39,40], partly as a consequence of the loss of cell-to-cell contact inhibition [41,42] and partly in response to developmental (e.g., fibroblast growth factor (FGF), platelet-derived growth factor-PDGF, and WNTs) and pro-inflammatory factors present in the lesion milieu and in the glial scar [2]. In the case of SCI, the OPC proliferative response is surprisingly long-lasting, being detected even at chronic stages [34]. An OPC proliferative burst is accompanied by a prominent reduction in neuron-to-OPC synaptic connectivity, as assessed by the transient disappearance of glutamatergic inputs onto virtually all proliferating OPCs responding to a demyelinating injury [43].

Newly born OPCs accumulate at the border of the lesion (e.g., at the stroke *penumbra*) together with astrocytes, whereas reactive microglia enter the margins of the fibrotic scar [44]. Such an OPC confinement depends at least, in part, on stromal cell-derived signals, as demonstrated by OPC infiltration in the central fibrotic core of the lesion in the case of fibroblast experimental ablation [45]. Acutely, OPCs produce fibronectin and laminin that protect axon growth cones from the neuroinflammatory milieu [46]. However, in the long term, glial cells overexpress matrix metalloproteases (MMPs), resulting in the degradation of hyaluronan and other ECM components into low molecular weight fragments that can amplify inflammation [47]. Glial cells, including OPCs, further modify the ECM composition by overexpressing chondroitin sulphate proteoglycans (CSPGs), among which brevican, neurocan, versican and NG2 are highly represented and known for contributing to a hostile environment and to axonal regrowth and neuronal migration [48].

On the one hand, most of the newly generated OPCs entrap axon growth cones and hamper regeneration; on the other hand, they do not differentiate into myelinating oligodendrocytes and do not contribute to remyelination. An increased number of immature OPCs and immature oligodendrocytes was reported at the border of the lesion, even at later times after stroke, SCI or a cortical stab wound [35,36,49], suggesting a blockade in their progression along the lineage or a local requirement for cells exerting roles other than differentiation into myelinating cells. The first hypothesis is corroborated by the presence of a plethora of environmental signals in the lesion milieu that operate as inhibitors of OPC differentiation, including pro-inflammatory cytokines and CSPGs (Figure 1) [50,51,52]. Accordingly, when plated on CSPG-coated plates, OPCs show maturation delay, reduced process outgrowth, and impaired migration, suggesting a direct chemorepulsive effect that alters their potential to access demyelinated sites and the consequent recovery process [53]. Similar repellent effects of CSPGs in oligodendrocyte function were observed in scars of traumatic origin. After SCI in rats, treatments with chondroitinase increased the numbers of OPCs surrounding lesions in the first two weeks post-lesion and the number of OPCs inside the lesion without altering OPC proliferation and cell death, likely as a consequence of improved OPC migration. OPC migration into lesioned sites was shown to be necessary to enable the increased axonal sprouting and improved recovery observed in the second week after SCI [54,55]. Interestingly, CSPG-induced oligodendrocyte maturation impairment could not be reverted by previously reported pro-remyelinating compounds such as clemastine, benztropine, and miconazole, which suggests that remyelination strongly relies on the extracellular microenvironment [56,57,58]. Furthermore, OPCs have functional receptors for many cytokines, such as IL-10, IL-6, IL-4, IL-18, IFN-γ and TNF-α [50,51,52]. These factors, primarily secreted by astrocytes and microglia, were shown to have a negative impact on OPC maturation both in vivo and in vitro [59,60].

OPC or oligodendrocyte transdifferentiation in other cell types may also contribute to such a failure in providing new myelinating elements. Specifically, fate-mapping experiments revealed that a fraction (10–25%) of OPCs acquire features of astrocytes (e.g., expression of the glial cell fibrillary protein (GFAP), or the Cx43, Aqp4, and Kcnq4 (Potassium Voltage-Gated Channel Subfamily KQT, Member 4) mRNAs and ion currents pattern similar to that observed in cortical astrocytes) upon cerebral stroke, cortical stab wound, SCI and experimental autoimmune encephalomyelitis (EAE) [61,62,63]. These OPC-derived astrocytes are a transient cell population characterised by a high expression of proliferation and motility markers [61]. As astrocytes have a limited capacity to migrate and amplify compared to OPCs (i.e., astrocyte numbers increase just by 10–20% after acute injury [64,65]), it has been proposed that OPC-derived astrocytes might temporarily perform the functions of astrocytes (such as regulating extracellular ion and neurotransmitter homeostasis or providing an energy supply) in proximity to the lesion [61], and then die or re-enter the oligodendroglial lineage [62]. Unexpectedly, a subpopulation of mature oligodendrocytes has also been shown to activate astrocytic genes and transgress via a transitional precursor phenotype toward the astroglial fate after acute brain injury [66]. Mechanistically, mature oligodendrocyte-to-astrocyte conversion has been shown to be microglia-dependent and mediated by the activation of the IL-6 signalling, likely acting in concert with other cytokines such as LIF and BMP4 [66]. Although the specific function of this additional population of astrocytes remains to be determined, it is tempting to speculate that these oligodendrocyte-derived astrocytes could exert a neuroprotective and pro-myelinating role [67,68], thereby opening promising avenues for the treatment of acute brain injuries.

In addition, after injury, NG2 cells can trans-differentiate into Schwann cells. Yet, the appearance of NG2 cell-derived Schwann cells is context-dependent (i.e., they are detected following SCI but not after a cortical stab wound injury), and their actual contribution to remyelination remains controversial [69,70].

In agreement with the idea that, upon injury, OPC roles go beyond the generation of new myelinating cells, recent studies have shown that OPCs per se participate in tissue remodelling and healing. Although OPCs were shown to contribute to synapse and axon pruning during normal development, they were also shown to selectively internalise myelin debris through phagocytosis upon injury [32,59,71,72]. Moreover, in the acute phase of white matter injury upon prolonged cerebral hypoperfusion, metalloproteinases (e.g., MMP9) released specifically by OPCs promote blood–brain barrier (BBB) leakage and infiltration of blood-derived cells at the lesion site [73]. Moreover, the ablation of proliferating NG2 glia results in reduced astrocyte hypertrophy and disorganisation of the glial scar, which eventually leads to delayed brain wound closure and worse functional outcomes [44,74]. Similarly, upon an inflammatory challenge, OPC depletion leads to a profound downregulation of the expression of microglia-specific genes and an exacerbated inflammatory response [75]. Consistently, manipulations of OPCs not resulting in their depletion (e.g., OPC-specific deletion of β-catenin) reduce astrocyte hypertrophy and GFAP expression and promote the microglia/macrophage acquisition of an anti-inflammatory/pro-regenerative phenotype [76]. Such effects likely depend on OPCs’ ability to release cytokines and chemokines and to present antigens when exposed to inflammatory conditions [77]. Both these capabilities are shared also by more advanced stages along the oligodendroglia lineage. For instance, immature oligodendrocytes can upregulate the expression of toll-like receptor TLR3, IL-1β, IFN-β, Ccl2, and Cxcl10 when stimulated with IL-1β [78]. Similarly, the chronic activation of NF-kB, the transcription factor downstream to TNF-α, IL-1 and toll-like receptors [79], leads to exacerbated neuroinflammatory conditions in mature oligodendrocytes [80]. Moreover, recent transcriptomic data allowed the identification of populations of disease-associated mature oligodendrocytes enriched in immune-related and antigen-presentation genes (e.g., major histocompatibility complex -MHC- class II) in the brain of mouse models and Alzheimer’s disease and MS patients [28,59,81]. Together, this evidence suggests that, upon injury, oligodendroglia can actively participate in fuelling inflammation and might have a role in disease development and antigen presentation.

Yet, recent studies have pointed out a remarkable level of heterogeneity in the oligodendroglia—and particularly in OPC—response to injury as related to their regional distribution (e.g., grey matter vs. white matter; brain vs. spinal cord; dorsal vs. ventral forebrain/spinal cord). For instance, grey matter OPCs show a higher regenerative/reparative potential compared to white matter OPCs in MS lesions [82]. Moreover, in the forebrain, dorsal OPCs display a higher vulnerability—but also a higher regenerative response—compared to ventral OPCs [83,84]. Mature oligodendrocyte subsets with a spatial preference and distinct susceptibility have also been recently reported following traumatic SCI [83]. In a few cases, such heterogeneity has been associated with cell-intrinsic features (i.e., OPC developmental diversity [84,85]). Yet, it is more than likely that at least part of the regional differences observed in oligodendroglia behaviour upon injury depends on their microenvironment, i.e., on the OPC/oligodendrocyte interaction with astrocytes, microglia and blood vessel-associated cells endowed with distinct features, as well as with an ECM with a different composition at distinct sites. While this idea still waits an experimental validation in pathological conditions, data obtained in the intact CNS are in line with this view, showing, for instance, that white matter astrocytes are less supportive for myelination compared to those in the grey matter, and that the higher responsivity of white matter OPCs to PDGF depends on their interaction with local microglia [82]. Of note, regional differences, as related to the oligodendroglia interaction with the “local niche”, may potentially impact on the design of treatments aimed at sustaining oligodendroglia functions upon injury at distinct locations (see below).

## 5. Therapeutic Approaches and Targets for Promoting Remyelination and Lesion Resolution

In the field of neurodegenerative disorders characterised by glial scar formation and demyelination, the main goal of treatment is to recover damaged motor and sensory axons, promote remyelination, and heal the glial scar. More recently, remyelination has increasingly emerged as an important strategy to protect axons from degeneration also after acute injuries. In the last decade, three main approaches have been proposed for improving remyelination: cellular transplantation, enhancement of cell transdifferentiation toward the oligodendroglial fate and the promotion of OPC differentiation by fostering pro-myelinating mechanisms and/or inhibiting pathologically altered mechanisms.

### 5.1. Cell Replacement Strategies

**Figure 2:**
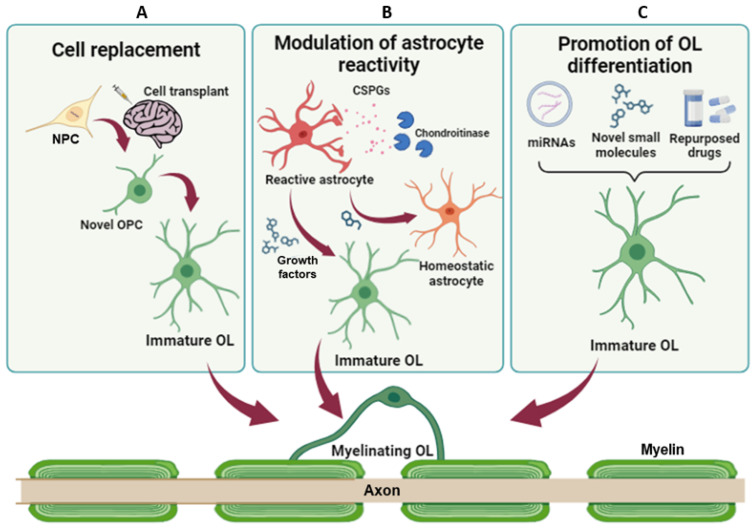
Therapeutic strategies to promote remyelination and lesion resolution. (**A**) NPC transplantation has the potential to restore myelination and neuronal connections, replacing damaged cells. (**B**) Modulation of astrocyte reactivity and promotion of their transdifferentiation toward the oligodendroglial fate can reduce the levels of inhibitory factors, promoting axon regeneration and endogenous remyelination. (**C**) Promotion of OPC differentiation and/or inhibition of pathologically altered mechanisms aim at fostering endogenous remyelination and, in turn, protect axons from damage. NPC = Neural progenitor cell; OPC = oligodendrocyte precursor cells; OL = oligodendrocyte; CSPG = chondroitin sulphate proteoglycans.

Cell transplantation emerged as one of the most effective strategies, with the ability to potentially restore functionality and neuronal connections by replacing damaged cells. Grafted cells could provide trophic support for neurons and manipulate the environment within the lesion to facilitate axonal regeneration, promote plasticity and foster endogenous remyelination (Figure 2A) [86]. Sources for cell transplantation are typically multipotent stem cells, including embryonic stem cells (ESCs), neural stem cells (NSCs) and neural precursor cells (NPCs), including directly reprogrammed NPCs, induced pluripotent stem cells (iPSCs) and mesenchymal stem cells (MSCs) [87].

In the context of SCI, several studies have highlighted the relevance of remyelination by graft-derived cells for neurobehavioral recovery after transplantation [88,89]. Following these findings, Nori and colleagues have developed a novel method to directly generate reprogrammed NPCs committed toward the oligodendrogenic fate (oNPCs) [90] to use them in an integrated strategy employing a cross-linked methylcellulose (XMC) hydrogel containing chondroitinase ABC (ChABC), the enzyme responsible for the degradation of the sulphated glycosaminoglycan chains on the CSPGs [91]. Indeed, previous studies have reported therapeutic efficacy for NPC transplantation in the subacute period, but effective therapies to target the chronic phase of SCI are greatly lacking due to the unfavourable microenvironment of the chronically injured spinal cord [92,93]. Furthermore, oNPC transplantation in combination with intrathecal injection of XMC-ChABC increased the survival rate of the grafted cells 12 weeks post-treatment. Grafted cells mainly differentiated into mature CC1-positive oligodendrocytes, contributed to the formation of mature myelin sheaths on spared axons and promoted the formation of nodes of Ranvier. More importantly, XMC-ChABC and oNPC combinatorial therapy improved motor function after chronic SCI, compared to the other groups [90], representing a clinically promising option to regenerate the chronically injured spinal cord.

The advent of gene therapy allowed the delivery of stem cells expressing therapeutic genes to increase the viability of damaged cells and promote regeneration while also limiting inflammation, demyelination and astrogliosis. In this context, Park and colleagues recently tested the possibility of increasing functional recovery from SCI by transplantation of NPCs overexpressing human arginine decarboxylase (hADC) [87], the enzyme that synthesises agmatine [94], an endogenous primary amine with neuroprotective effects [95]. Six weeks after SCI, the hADC–NPC transplantation improved neurological outcomes, induced oligodendrocyte differentiation and remyelination, increased neural lineage differentiation, and decreased glial scar formation [87]. Moreover, locomotor and bladder function were both recovered, suggesting that hADC overexpression in NPCs is a potential therapeutic approach for SCI treatment.

NPC-based therapies may also represent a valuable option for the treatment of progressive MS (PMS). In the EAE model, transplanted NPCs showed pathotropic properties, migrating to demyelinating areas and inducing a rescue of the functional impairment in transplanted rodents [96]. Within these areas, OPCs markedly proliferated and differentiated, with many of them being of donor origin and actively remyelinating axons. Furthermore, a significant reduction in astrogliosis and a marked decrease in both the extent of demyelination and axonal loss were observed in transplanted animals. NPC transplantation ameliorated EAE in a non-human primate model as well [97]. Very recently, results from a phase I clinical trial involving 12 severely disabled people with PMS, who had already received the available therapies with little or no success, have demonstrated the safety and tolerability of NPC transplantation in humans [98]. This study has also shown a significant reduction in the loss of cerebral tissue, evaluated by magnetic resonance imaging during the subsequent two years, and an amelioration of the CSF profile in the patients who received the higher dose of NPCs [98]. Further clinical studies in a larger cohort of patients are necessary to establish whether NPC transplantation might have a long-lasting bystander effect in PMS patients.

Beyond NPCs and NSCs, oligodendrocytes have been considered suitable therapeutic targets for cell replacement therapy in neurodegenerative diseases. In this respect, a recent study evaluated the contribution of the inhibitory microenvironment combining glial scar ablation (GSA) with the transplantation of iPSC-derived ventral spinal neural progenitor (pre-OPCs) [99], a cell population that arises from the motor neuron progenitor domain in the developing neural tube, which is able to generate both motor and interneurons and subsequently OPCs [100]. In SCI, the magnitude of the lesion cavity was significantly decreased when GSA was performed after pre-OPCs injection when compared to the GSA-induced-only and pre-OPCs-injected-only groups. Grafted cells survived and differentiated into astrocytes, oligodendrocytes and neurons when transplanted into chronically injured rats. The local environment created by GSA addressed the transplanted cells towards oligodendrocyte lineage differentiation at the expense of ventrally located V2a interneurons [99]. Altogether, these observations highlight the importance of the crosstalk between transplanted cells and local cues from the host environment to drive the successful integration and differentiation of xenografts. The mentioned studies that applied cell replacement strategies are summarised in Table 1.

### 5.2. Limiting Astrogliosis to Favour Remyelination

Acutely, astrogliosis does limit the spread of inflammation, but, later, it creates a physical and chemical barrier to the regeneration and axon remyelination. Despite many therapies for CNS injury seeking to remove this barrier by blocking astrogliosis, several studies suggested that, in certain contexts, blocking reactive astrocyte formation impedes functional recovery [101]. Therefore, rather than halting astrogliosis entirely, alternative therapies could target the specific factors released by reactive astrocytes (e.g., CSPGs) that inhibit axon regeneration and remyelination (Figure 2B). Astrocytes in the white matter of MS lesions associate with CSPGs, and remyelination tends to be more robust in areas with less reactive astrocytes, lower levels of CSPGs and a higher number of OPCs. The mechanisms by which CSPGs build a hostile environment for recovery from glial scarring are still unclear. CPSGs can bind and activate different receptors, such as the protein tyrosine phosphatase-sigma receptor family (PTPR) [102], mostly known to regulate synapse formation. When activated by CSPGs, it activates the RhoA-ROCK signalling, which has been associated with impaired oligodendrocyte maturation and myelination by inhibiting the extension and branching of oligodendrocyte processes [103]. In vitro, silencing of PTPRσ reversed the inhibitory effect of CSPGs on oligodendrocyte process outgrowth and myelination. A similar effect on oligodendrocyte process outgrowth and myelination was observed using Y-27632, an inhibitor of ROCK [102]. Inhibiting this pathway using the intracellular sigma peptide was also shown to reduce gliosis and demyelination in the LPC model and to improve oligodendrocyte remyelination in the optic nerve after LPC treatment [104]. Another recent study in a mouse model of MS [105] described a pharmacological strategy aimed at reducing CSPG deposition into the lesions by administering UCM03025, a new potent selective and reversible inhibitor of monoacylglycerol lipase (MagL), the enzyme that accounts for 2-arachidonoylglycerol (2-AG) degradation. The 2-AG accumulation reduced microglia activation, CSPG levels and astrogliosis around demyelinated lesions in the spinal cord of Theiler’s murine encephalomyelitis virus-infected mice. Moreover, inhibition of 2-AG hydrolysis increased the number of mature oligodendrocytes, leading to remyelination and functional recovery. These data suggest that targeting MAGL represents a promising therapeutic strategy for the treatment of progressive phases of chronic demyelinating diseases and other pathologies involving white matter injury.

Accumulating evidence shows that, in pathological tissues, reactive astrocytes have stem cell properties and multiple differentiation potentials [106], suggesting that some extracellular signals in the pathological microenvironment are involved in cell fate switching. Thus, directly reprogramming or converting astrocytes to neurons and oligodendrocytes has been investigated as a potential therapeutic strategy that aims at attenuating astrogliosis and facilitating brain and spinal cord remyelination (Figure 2B). A recent study showed that reactive astrocytes could be addressed toward the oligodendroglial lineage by the addition of Neuregulin1 (Nrg1), an extracellular axon-localised factor able to promote oligodendrogenesis and remyelination [107]. Cultured reactive astrocytes treated with Nrg1generated oligodendrocyte-like cells, characterised by the expression of oligodendrocyte lineage markers (i.e., PDGFR-α and O4). In addition, the number of cells co-expressing PDGFR-α or O4 or CNPase and GFAP was significantly increased in the injured spinal cord treated with Nrg1 for 2–4 weeks, compared to vehicle-treated mice [108]. More importantly, in the Nrg1-treated group, demyelination was significantly lower compared to the controls, suggesting that Nrg1-promoted astrocyte-to-oligodendrocyte conversion may contribute to enhanced remyelination after SCI [108]. Further characterisation of the extracellular factors in the lesion microenvironment will not only help to reveal the molecular mechanism underlying the astrocyte-to-oligodendrocyte fate change but will also aid in identifying therapeutic targets for improving the efficiency of lineage conversion.

Conversion of reactive astrocytes to other cell types can also be achieved by somatic reprogramming techniques. A recent study has shown that SCI elicits the neurogenic potential of OPCs and that SOX2—a transcription factor that plays a prominent role in neurogenesis—is sufficient for neurogenic reprogramming of these cells, which can eventually lead to functional neurogenesis in a non-neurogenic adult mouse spinal cord [109]. Although this injury-induced phenotypic reprogramming was found to be transient and incomplete for producing mature neurons, the injection of SOX2-producing viral particles into the surrounding lesion area increased neurogenesis, reduced glial scar volume and ultimately contributed to functional improvements after SCI [109].

Among the different transcription factors involved in OPC specification and differentiation, SOX10 is one of the most investigated and widely used to produce inducible oligodendrocytes from fibroblasts, iPSCs and NSCs [110,111,112]. SOX10 is also involved in the transdifferentiation of astrocytes to oligodendrocytes occurring in SCI, and this conversion can be enhanced by EGF-signalling activation [113]. Continuous delivery of EGF into the injury area after SCI significantly increased the percentage of SOX10-induced oligodendrocytes, compared to untreated mice, whereas EGFR inhibitor Gefitinib counteracted the synergistic effect of EGF and SOX10. Similarly, Gefitinib reduced the transdifferentiation in the SOX10-only infected tissues as well, suggesting that, in the pathological milieu, EGF participates in the astrocyte-to-oligodendrocyte reprogramming [113]. The mentioned studies targeting astrocytes to improve remyelination are summarised in Table 2.

### 5.3. Promoting OPC Differentiation

Remyelination is controlled by both intrinsic and extrinsic factors that act either as inhibitors or promoters of OPC differentiation [114,115]. As discussed above, the incomplete remyelination reported in MS and other neurodegenerative disorders is partly due to the failure of OPCs to differentiate into myelinating oligodendrocytes rather than to a reduction in the pool of OPCs. Therefore, new therapeutic agents promoting OPC differentiation and remyelination are urgently needed [116]. Molecules able to promote remyelination can be classified based on their nature [117] in small molecules targeting physiological or pathologically altered receptors (e.g., GPCRs and intracellular receptors), endogenously occurring free molecules (e.g., microRNAs, metabolite and peptides), and non-physiologically occurring free molecules (e.g., synthetic compounds and plant-derived compounds) (Figure 2C). In this paragraph, we provide and discuss some examples for each category.

Among the different GPCRs expressed by the oligodendrocytes during their differentiation, GPR17 and GPR37 are known for their role in preventing excessive or premature myelination via a cAMP-dependent mechanism. GPR17, whose expression starts in early OPCs, reaches its maximal expression in pre-oligodendrocytes and must be downregulated to allow terminal maturation [118,119]. Previous studies have shown an increased number of GPR17-expressing cells blocked at immature stages in several models of neurodegenerative diseases [120,121] and human brain pathological specimens [122]. Accordingly, GPR17 KO induced untimely oligodendrocyte maturation, while its overexpression inhibited oligodendrocyte development and myelination [123,124]. GPR17 has been explored as a potential therapeutic target in remyelination, either by inhibiting GPR17 via antagonists or by activating the reserve pool of GPR17-expressing OPCs to promote their maturation [125].

Instead, GPR37 starts to be expressed later, in pre-oligodendrocytes, and its expression is maintained in more mature stages. Like GPR17, GPR37 stimulation reduces the cAMP levels, while its inhibition increases the cAMP levels and the Epac/Raf/ERK downstream signalling. A recent study found that osteocalcin, a hormone synthesised in osteoblasts, can cross the BBB and interact with GPR37 to inhibit the transition between pre-oligodendrocytes and myelinating oligodendrocytes [126]. Another GPCR expressed by astrocytes and OPCs [127], called GPR37L1, shares structural and functional similarities with GPR37 and plays an important role in brain health and remyelination as well. Both receptors are activated by prosaposin [128], a precursor of saposins, which is involved in the metabolism of sphingolipids, a fundamental building block of the myelin sheath. Prosaposin (PSAP) and prosaptides (peptides mimicking the neurotrophic region in prosaposin) have been shown to protect oligodendrocyte cell lines and stimulate myelin lipid synthesis [129,130]. Interestingly, a recent spatial transcriptomic analysis of neurodegeneration in PMS indicated that the interaction between neuronal PSAP and oligodendroglial GPR37L1 is reduced in disease conditions and highlighted this receptor as a potential novel drug target for progressive MS [131]. Further studies are necessary to clarify the exact role of GPR37/GPR37L1 in remyelination.

Another receptor, called GPR149, recently found to be highly expressed in OPCs and downregulated during oligodendrocyte differentiation, acts as a negative regulator of myelination and remyelination in the CNS [132]. Despite the signal transduction mechanism and endogenous ligands of GPR149 still being unknown, its inhibitory action on oligodendrocyte differentiation is likely mediated by the activation of the ERK1/2 pathway. Similarly to GPR17, genetic deletion of GPR149 in mice resulted in the transiently precocious differentiation of oligodendrocytes and enhanced myelination during development and myelin regeneration following cuprizone-induced demyelination [132], suggesting that blocking GPR149 might be an intriguing way to promote myelin repair in demyelinating diseases.

Repurposing approaches allowed the identification of clobetasol and miconazole, marketed drugs with a history of safe use, as promising remyelinating molecules that are able to markedly improve motor function, reduce demyelination and improve MBP expression in the spinal cord when administered daily in EAE mice starting from the peak of disease [133]. Despite this promising evidence, their effect on SCI has not been investigated yet. The protective effect of miconazole has been confirmed following premature infant cerebral white matter injury and peripheral nerve crush injury [134,135]. A following repurposing study focused on drugs acting on the nucleocytoplasmic shuttling of the p57kip2 protein, an early cellular process involved in OPC differentiation decision [136]. After evaluation in human primary OPCs and in organotypic cerebellar slice cultures, the steroid danazol and the anthelmintic parbendazole were selected for an in vivo evaluation [137]. Of these, parbendazole increased the number of mature oligodendrocytes and enhanced spontaneous remyelination following cuprizone-induced demyelination, providing a new pharmacological compound with the potential to boost the myelin repair processes. Of note, another member of the benzimidazole-based anthelmintic family, namely flubendazole, has shown pleiotropic effects in the SCI model, improving locomotor function and tissue sparing and reducing B-cell proliferation and astrocyte activation [138]. The possible direct effect of flubendazole on oligodendrocytes has not been investigated yet.

Other FDA-approved drugs that showed promising results in demyelinating models are montelukast and clemastine. Montelukast is a CysLT1R and GPR17 antagonist, approved as adjuvant therapy for asthma in both children and adults. Montelukast’s neuroprotective and anti-inflammatory effects have been proven in different models of neurodegenerative diseases, including SCI, MS, stroke, amyotrophic lateral sclerosis and Huntington’s [139,140,141,142,143,144]. Montelukast’s beneficial effects are likely mediated by its multiple actions on microglia/astrocytes and oligodendrocytes, expressing CyLT1R and GPR17, respectively. Clemastine is a first-generation H1 histamine antagonist with anticholinergic properties that has been shown to be effective in promoting myelination in neonatal hypoxia and SCI models [145,146]. A phase 2a clinical trial in patients with relapsing-remitting MS is now evaluating the beneficial effects of clemastine in combination with metformin, a drug that is already used to treat type 2 diabetes, which showed remyelinating properties [147,148,149]. Other studies suggested that clemastine modulates the behaviour of other glial cells. In a mouse model of depression, clemastine significantly suppressed microglial M1-like activation and improved hippocampal astrocytic loss [144]. Of note, a recent paper also demonstrated that clemastine reduces the density of CD11c + microglial cells, a transient population involved in developmental myelination, suggesting that clemastine’s final outcome on myelination may depend on microglia–oligodendrocytes crosstalk [150]. The mentioned studies exploiting already-approved drugs to improve remyelination are summarised in Table 3.

In the last decade, the role of microRNAs in oligodendrocyte development and the consequences of their dysregulation in these cells have also been extensively investigated, thus opening the way to exploit miRNA mimics or antago-miR as pharmacological targets [151,152]. Among the numerous miRNAs able to directly influence oligodendrocyte development, miR-219 and miR-338 are the most characterised. These oligodendrocyte-specific miRNAs promote oligodendrocyte differentiation by repressing negative regulators of oligodendrocyte differentiation [153,154]. Administration of exogenous miR-219 and miR-338 promoted myelin regeneration in models of demyelinating diseases [155,156,157,158]. In this context, a recent study evaluated the effects of miR-219/miR-338 delivery on microglia and astrocyte behaviour, showing that their overexpression diminished microglial expression of pro-inflammatory cytokines and suppressed astrocyte activation [159]. These results suggest that miR-219/miR-338 CNS delivery represents a promising treatment for axonal remyelination following nerve injuries.

miRNAs can act as inhibitors of oligodendrocyte differentiation. Overexpression of miR-125a-3p in cultured OPCs impaired oligodendrocyte maturation, while administration of an antago-miR promoted maturation through different mechanisms [160]. Expression levels of this miRNA have been found to increase following demyelinating injuries in MS animal models and brain specimens, suggesting its possible involvement in disease pathogenesis. Accordingly, the in vivo silencing of miR-125a-3p following lysolecithin-induced demyelination promoted myelin repair [161]. Due to their unique interacting features, miRNAs can act at different stages of a process on different targets, leading to different outcomes. This implies that potential off-target effects can occur in miRNA-based therapy. It is the case of miR-146a, a miRNA, that it recently showed therapeutic potential for remyelination. Several studies demonstrated that miR-146a was able to increase oligodendrocyte differentiation and remyelination in animal models [162,163,164]. However, further studies indicated that miR-146a-deficient mice demonstrated increased oligodendrocyte numbers and reduced inflammation, demyelination and axonal loss following cuprizone-induced demyelination [165]. This example highlights the importance of designing stage-specific and cell-specific therapeutic strategies based on miRNA mimics/inhibitors to prevent unwanted off-target effects.

## 6. Conclusions

Due to the cellular heterogeneity and the complex dynamics of the lesions in time and space, therapeutic intervention to treat glial scar or scar-associated neuroinflammation still remains a challenge. It is clear that glial scar formation is part of a regenerative attempt and should not be completely blocked. Instead, the inhibitory factors that progressively establish a hostile environment should be dissected to identify more focused therapies. The roles of oligodendrocytes and OPCs have been overlooked in glial scars. Early after injury, oligodendrocyte apoptosis leads to demyelination, whereas OPCs react and migrate toward the damage to replenish and repair. Acutely, they form synapse-like connections with the tips of transected axons and produce ECM components that protect axon growth cones from the neuroinflammatory milieu. However, in the long term, they overexpress MMPs and CSPGs, contributing to the production of aberrant unfavourable ECM components and to the generation of a hostile environment, which in turn impairs long-term axonal regeneration and inhibits OPC differentiation and remyelination. The production of high amounts of CSPGs within the lesions is associated with the chemorepulsion and pathological behaviour of OPCs. NG2 is one of the major CSPGs expressed on the surface of early OPCs, whose accumulation at the lesioned areas could take part in a vicious cycle, impeding their own differentiation. Accordingly, the depletion of CSPGs was shown to successfully restore OPC differentiation and remyelination in several models of disease.

Fostering remyelination is an unmet need in the context of demyelinating diseases such as MS. However, since oligodendrocyte dysregulation and demyelination are pathological features of several neurodegenerative diseases or acute conditions, and often they take place in the early phases of neurodegeneration, it is possible to speculate that remyelination would also be a potential strategy in other disorders, trauma and brain ischemia. It is worth observing that the generation of a favourable microenvironment is a key step to paving the way to remyelination. Accordingly, cell transplantation approaches have shown promising results when coupled with other interventions aimed at reestablishing a favourable microenvironment, targeting CSPG deposition and degradation. These results may explain why previously reported pro-remyelinating compounds have shown poor efficacy in the presence of an unfavourable CSPG-rich environment, also suggesting that these therapeutic approaches should be complemented with other compounds targeting the hostile microenvironment.

## Figures and Tables

**Table 1:** Summary of the studies that applied cell replacement strategies to improve remyelination.

Disease Model	Transplanted Cells	Co-Intervention	Outcomes	Reference
SCI	OPC-committed NPCs	Chondroitinase ABC	Formation of nodes of Ranvier. Improvement in remyelination and motor functions.	[90]
SCI	NPCs	Overexpression of human arginine decarboxylase	Decreased glial scar formation. Increased remyelination and neuronal differentiation. Improvement in neurological outcomes.	[87]
MS (EAE)	NPCs	-	Decreased demyelination and astrogliosis. Increased remyelination and improvement in neurological scores.	[96]
SCI	Pre-OPCs	Glial scar ablation	Generation of new OLs and neurons.	[99]

**Table 2:** Summary of the studies targeting astrocytes to improve remyelination.

Disease Model	Treatment	Mechanism of Action	Outcomes	Reference
MS (LPC)	Intracellular sigma peptide	Inhibition of protein tyrosine phosphatase-sigma (PTPσ)	Decreased demyelination and astrogliosis Increased remyelination and functional recovery in the optic pathway.	[104]
MS (TMEV)	UCM03025	Inhibition of monoacylglycerol lipase (MagL)	Reduction in microglia activation, astrogliosis and CSPG levels around demyelinated lesions. Improvement in remyelination and motor functions.	[105]
SCI	Neuregulin1	PI3K-AKT-mTOR pathway	Reduction in astrogliosis. Enhancement of remyelination, axonal preservation and locomotor recovery.	[108]
SCI	SOX2	-	Reduction in glial scar volume. Increased neurogenesis and improvement in motor functions.	[109]
SCI	SOX10	Activation of EGF signalling	Increased generation of oligodendrocytes.	[113]

**Table 3:** Summary of the studies exploiting already-approved drugs to improve remyelination.

Disease Model	Treatment	Mechanism of Action	Outcomes	Reference
MS (EAE and LPC)	Clobetasol	Glucocorticoid receptor Signalling	Decreased myelin loss and T-cell activation. Promotion of OPC maturation and remyelination.	[133]
MS (EAE and LPC) PWMI PNCI	Miconazole	ERK1/2 and Nf-Kb activation	Decreased myelin loss and microglia M1 activation. Promotion of OPC maturation and remyelination.	[133,134,135]
MS (CPZ)	Parbendazole	ERK1/2 activation	Promotion of OPC maturation and remyelination.	[137]
SCI	Flubendazole	Inhibition of cyclin B and BTK pathways	Reduction in myelin loss, B-cell proliferation, and astrocyte activation	[138]
SCI, MS, Stroke, ALS and HD	Montelukast	Inhibition of CysLT1 and GPR17 receptors	Neuroprotective effect on permanent focal cerebral ischemia. Reduction of neuroinflammation and myelin loss. Promotion of OPC maturation and remyelination.	[139,140,141,142,143,144]
Neonatal hypoxia and SCI	Clemastine	Inhibition of M1 muscarinic receptor	Promotion of OPC maturation and remyelination.	[145,146]
